# Abiotic stress triggers electrical synchronisation of shoot and leaves in soybean plants: a clue for plant attention-like

**DOI:** 10.1080/15592324.2025.2577400

**Published:** 2025-11-05

**Authors:** Thiago Francisco de Carvalho Oliveira, André Geremia Parise, Helena C. Tasca, Douglas Posso, Gabriela Niemeyer Reissig, Gustavo Maia Souza

**Affiliations:** a Laboratory of Plant Cognition and Electrophysiology (LACEV), Department of Botany, Institute of Biological Sciences, Federal University of Pelotas, Pelotas, Brazil; b School of Biological Sciences, University of Reading, Reading, United Kingdom; c School of Agriculture, Policy and Development, University of Reading, Reading, United Kingdom; d Embrapa Clima Temperado, Pelotas, Brazil

**Keywords:** Plant electrophysiology, electrical synchronization, plant cognition, selective attention, stress response

## Abstract

Plants rely on sophisticated intercellular communication to coordinate systemic responses to environmental challenges. Electrical signals contribute for rapid, long-distance integration of plant parts. This study investigated how distinct stressors—localized injury (cutting and fire to a leaflet) and systemic salt stress (applied to the roots)—triggered electrical synchronization across different modules (stem and leaves) in soybean (Glycine max) plants. We continuously recorded variations of electrical potential from four plant modules before and after stress application. Time-series analyses, including Detrended Fluctuation Analysis (DFA), Approximate Entropy (ApEn), Fast Fourier Transform (FFT), and Power Spectral Density (PSD), were employed to characterize signal features. Inter-modular synchronization was then assessed by Pearson correlation of these derived features between the modules. The results indicate that different stressors modulate electrical synchronization between plant modules in distinct ways: while cutting and fire stress induce a more immediate and integrated response, showed as higher correlation between modules, salt stress promotes more gradual changes in signal dynamics. These findings reinforce the hypothesis that electrical signalling plays an important role in the functional integration of stress responses, and may indicate a possible attentional state in plants.

## Article highlight

Using a like-attentional state, plants electrically synchronize their modules to coordinate a fast, integrated response to both local and systemic stress.

## 1. Introduction

Plants are made of repeated, semi – autonomous parts (i.e. leaves, internodes and roots) in an essentially modular structure.^1^^,^^2^ However, plants do not behave merely as a collection of independent modules reacting to their local environment. Instead, they exhibit a high degree of coordination across the parts, dynamically integrating environmental information to produce coherent physiological and morphological responses as an emergent whole.^3^ This integration, for instance, is a key to allow complex behaviours such as systemic foraging.^2^^,^^4^

Modular plant integration relies on a rich internal communication network involving chemical, hydraulic, and electrical signals. Among these, electrical signals are particularly relevant for their rapid propagation across plant tissues via ion fluxes, enabling fast, long–distance coordination.^5^^,^^6^ Electrical signals have been shown to mediate early responses to a variety of environmental stressors, such as herbivory, light shifts, or salinity.^7-10^ The totality of these electrical activities, referred to as the plant electrome,^11^^,^^12^ reflects both baseline and stimulus–induced dynamics that can support the necessary integration to improve acclimation to environmental challenges.

From a systemic viewpoint, plants can be described as distributed information–processing organisms.^13^ Their modular architecture, while allowing local autonomy, also enables dynamic reconfiguration and coordination in response to environmental challenges.^14^^,^^15^ In this context, synchronised changes in the electrome across modules may indicate a temporary state of heightened internal coordination. Drawing inspiration from neuroscience, where attention is often correlated with transient synchronisation of neural oscillations across brain regions,^16^^,^^17^ we explore whether a similar principle may apply to plants.

In cognitive systems, synchronisation among distant processing units enhances signal fidelity and selective responsiveness to salient stimuli.^18^ Although plants lack neurons, their electrophysiological activity exhibits temporal structure, scaling properties, and stimulus–dependence–features commonly associated with integrative processing in biological systems.^12^^,^^17^^,^^19^ We hypothesise that the synchronisation of electrome activity among plant modules, especially following the perception of external stressors, represent a functional analogue to attentional states: a shift from decentralised autonomy to transient global integration that facilitates optimised responses.^16^^,^^17^^,^^20^

Based on this theoretical framework, we investigated how distinct abiotic stressors–local (cutting and fire) and systemic (salt stress)—influence the electrical synchronisation of different soybean (*Glycine max*) modules. To this end, we recorded electrome activity from the stem and different leaves simultaneously, before and after each stimulus, and applied multiscale time–series analyses (DFA, ApEn, FFT, PSD), followed by inter–modular correlation metrics.

## 2. Material and methods

As a well know plant model in electrome studies,^12^ soybean seeds (*Glycine max* (L.) Merril, TMG 2165 IPro cultivar) were grown under experimental controlled condition at the Laboratory of Cognition and Plant Electrophysiology (LACEV), in Federal University of Pelotas, Brazil. Plants were cultivated in 300 mL pots filled with organic commercial substrate (Beifort S10B, Beigrupo, Brazil) under artificial lighting (12-hour photoperiod, PPFD of 450 µmol m^−2^ s^−1^ , temperature of 25 °C ± 2). Irrigation was maintained daily to ensure water pot capacity, with full^21^ nutrient solution applied twice a week. The analyses were started when the plants reached stage V4 with three fully expanded leaves.^22^

**Figure 1.**
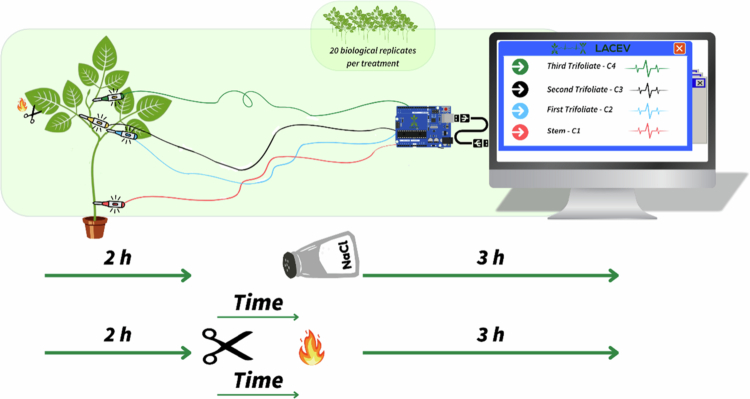
Schematic representation of the modularity experiment. Analyses were conducted on the base of the stem, as well as the first, second, and third trifoliate leaves. Cutting and fire stress were applied to the middle leaflet of the second trifoliate, while salt stress was applied to the root.

The electrical signals were recorded using non–polarised Ag/AgCl needle electrodes (EL452, Biopac Systems) inserted at specific plant regions (Figure 1) and connected to a four–channel data acquisition system (MP36; Biopac Systems; input impedance: 10 GΩ). A standard ECG protocol was employed at a 62.5 Hz sampling rate. To minimise noise from electrode insertion, plants were allowed to acclimate overnight before measurements. Electrome activities were collected for 2 hours under control conditions in order to settle a baseline,^23^ and for more 3 hours post–stimulus, capturing micro–voltage variations between electrodes spaced 1 cm apart as standard to avoid further variation to the signal.^23^ Analogue filters inherent to the system (0.05 Hz high–pass, 1.5 kHz low–pass) were applied during acquisition, followed by a 5th order digital Butterworth low–pass filter to further reduce residual noise and ensure precise feature extraction.

• Channel 1 (C1): base of the stem, 2 cm above the soil surface.
• Channel 2 (C2): petiole of the first trifoliate leaf.
• Channel 3 (C3): petiole of the second trifoliate formed during plant growth.
• Channel 4 (C4): petiole of the third trifoliate formed during plant growth.

The plants were divided and subjected to two types of stress. One group underwent salt stress through the application of 100 mL of salt solution (150 mM NaCl) directly to the substrate 30 seconds after the start of electrical signal capture following the 2-hour control period. The second group underwent cutting immediately followed by fire stress, where approximately 2 cm of the middle leaflet of the second trifoliate (C3) was cut with scissors, followed by exposure to a flame 1 cm away from the tip of the central leaflet of the second leaf, 60 seconds after the start of signal capture (based on^24^). Twenty biological replicates were performed for each condition.

The analyses of the electrical signal time series employed the following techniques:

1. Fast Fourier Transform (FFT): The Bluestein’s algorithm^25^ and the rfft Hermitian–symmetric algorithm from the scipy library^26^ used to compute the frequency components efficiently.
2. Power Spectral Density (PSD): PSD estimation was performed using the Welch method^27^ which divides the data into overlapping segments, calculates the periodogram for each, and averages the results. This analysis was implemented with the scipy library,^28^ using 4-second windows for the Welch method.
3. Detrended Fluctuation Analysis (DFA): DFA calculates a power–law scaling exponent, like the Hurst exponent, which quantifies long–term correlations in a time series. This method assesses the autocorrelation of a time series and provides insights into its long–term and short–term memory indices.
4. Approximate Entropy (ApEn): ApEn measures the level of organisation or regularity in a time series by considering the temporal order of its data points. This makes it particularly suitable for assessing the randomness or regularity of biological signals​​​​​​.^29^^,^^30^
5. Lyapunov Exponent (Lyap): The Largest Lyapunov Exponent (LLE) was calculated to assess the level of chaos and sensitivity to initial conditions within the electrophysiological time series, where positive LLE values are indicative of such dynamics. The estimation was performed using the Python library nolds. Specifically, the algorithm developed by Rosenstein et al., ^31^ was employed to estimate the LLE. The calculations were performed using the default parameters provided by the nolds library for this function.

To visualise these analyses, the Time Dispersion Analysis of Features (TDAF) method was applied.^24^ In this approach, the time series were divided into shorter segments (Each time series is divided into interchangeable one–minute segments, with a 10% overlap between cuts), preserving temporal information and associating each segment with corresponding features. These features were then aggregated for each time interval, enabling a dispersion analysis through metrics such as maximum, minimum, median, and quartiles. By plotting the analyses chronologically, the dynamic changes in feature dispersion over time were revealed.

Finally, Pearson's correlation method was used to calculate the relationships between the TDAF time series of all modules before and after the stress event in order to infer synchronisation between modules allowing to test our former hypothesis.

## 3. Results

### 3.1. Cut & fire–local stress

**Figure 2.**
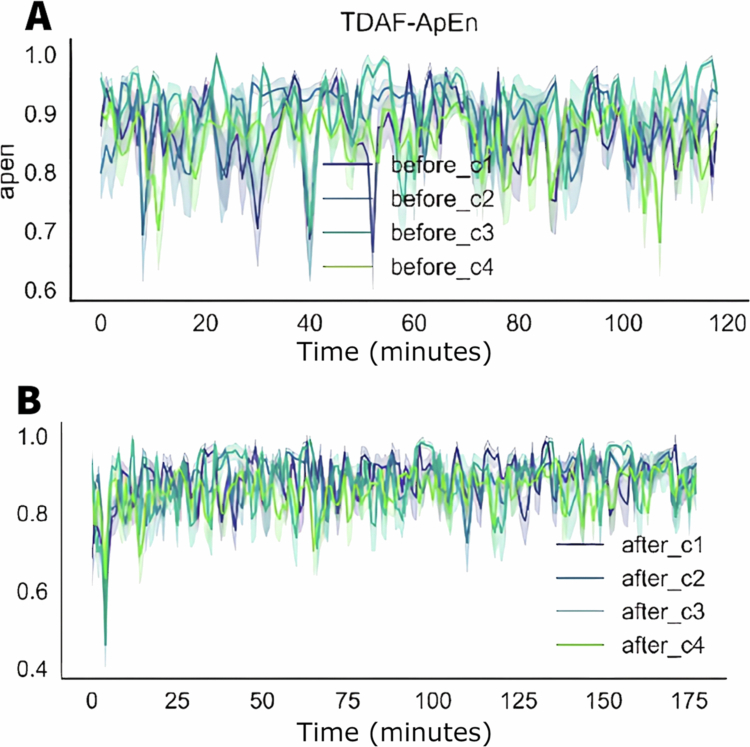
TDAF plot displaying ApEn values. The (A) panel shows measurements taken 120 minutes prior to the infliction of cutting and fire to the middle leaflet of the second trifoliate (C3). The (B) panel presents values measured 180 minutes after stress infliction. Dark lines represent the median values for each minute, and shaded areas indicate the maxima and minima values recorded at each minute. The analysis includes the following modules: Shoot (C1), first leaf (C2), second leaf (C3), and third leaf (C4).

Figure 2 shows the analysis of ApEn in the four modules before (as a control baseline) and after the stress. We noticed a low dispersion in both conditions indicating a more uniform distribution between the plants analysed. After the application of stress, a dip was observed within the first minute, with the lowest recorded value being 0.4. Subsequently, the values returned to the range observed prior to the stress event, but with reduced variation. Previously, the values fluctuated between 0.6 and 0.9 on average. Following the post–stress peak, the values stabilised, oscillating between 0.8 and 1 on average.

**Figure 3.**
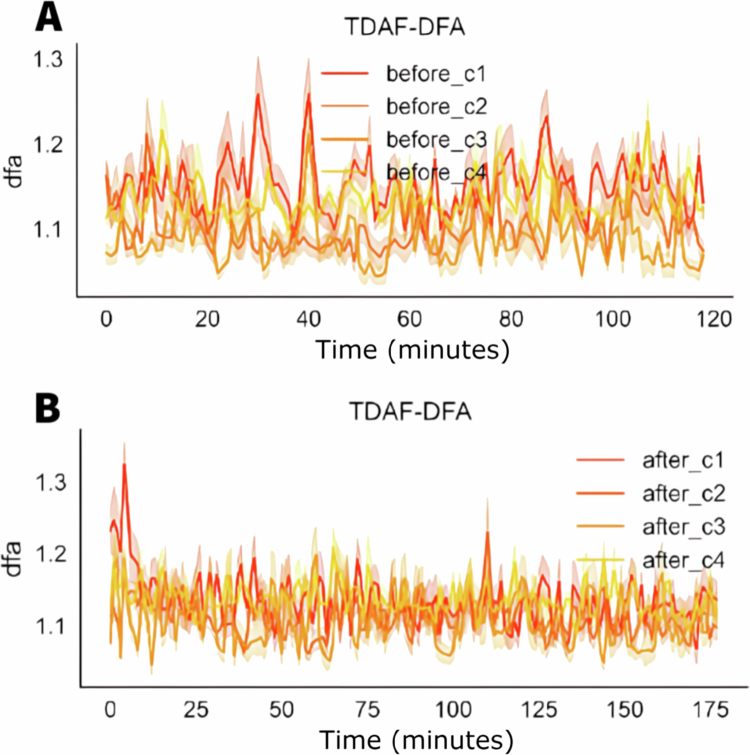
TDAF plot displaying DFA values. The (A) panel shows measurements taken 120 minutes prior to the infliction of cutting and fire to the middle leaflet of the second trifoliate (C3). The (B) panel presents values measured 180 minutes after stress infliction. Dark lines represent the median values for each minute, and shaded areas indicate the maxima and minima values recorded at each minute. The analysis includes the following modules: Shoot (C1), first leaf (C2), second leaf (C3), and third leaf (C4).

Figure 3 shows the graphs for the values of the detrended fluctuation analysis (DFA) through time, before and after the application of stress of cut and fire. Before the stress, the values of DFA show a greater variation, with significant spikes throughout the 120 minutes of the observation. After the stress, there was an initial increase in the DFA values in all the modules, with smaller variation throughout time and spikes less pronounced when compared to the values before the stress. The recovering after the initial increase shows that the DFA values stabilise rapidly aligning the values from all modules more tightly.

**Figure 4.**
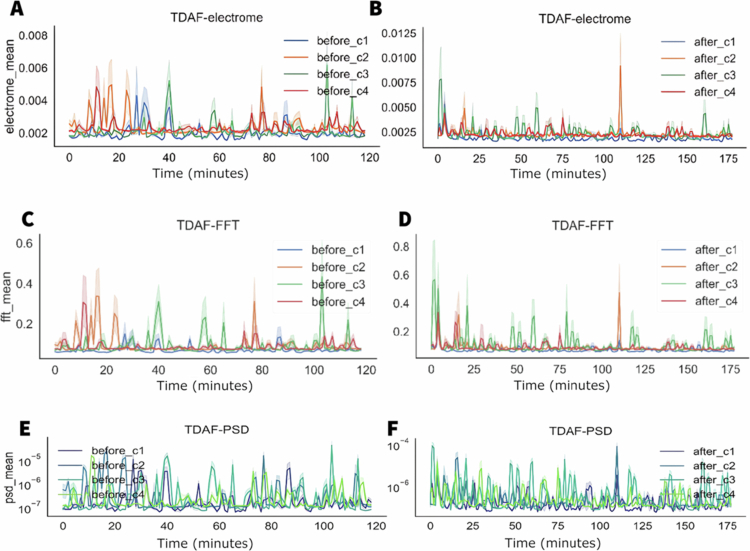
TDAF plot showing the average values for electrical potential difference (the electrome) (A, B), frequency (C, D), and PSD (E, F). The A, C, and E panels display measurements taken 120 minutes before the middle leaflet of the second trifoliate (C3) was subjected to cutting and fire stress, while the B, D, and F panels show measurements taken 180 minutes after stress application. Dark lines represent the median values at each minute, while the shaded areas indicate the minimum and maximum values. The analyses consider the following modules: Shoot (C1), first leaf (C2), second leaf (C3), and third leaf (C4).

In the first row of graphs in Figure 4, we can find the results for the electrical potential difference (the electrome) averaged for each minute. To the left, the data measured before the stress, we can find the incidence of a greater variation in the activity, demonstrated by the presence of several spikes specially in the first 20 minutes. To the right, the data obtained after stress, we can see a significant 10-fold increase in the electrical potential on average. However, there is a trend towards a smaller variation of activity and a synchronisation between the modules.

In the second row, we can find the values of frequency in average. Again, we can observe a behaviour like the electrical potential difference. Before the stress, there is a higher incidence of variation with more prolonged spikes, and after the stress, a decreased variation and synchronisation except for some spikes measured in the second leaf (C2), the leaf that was injured. The PSD data showed a higher variation in energy after the stress. The tree analyses indicated the moment of stress as the beginning of the time series.

**Figure 5.**
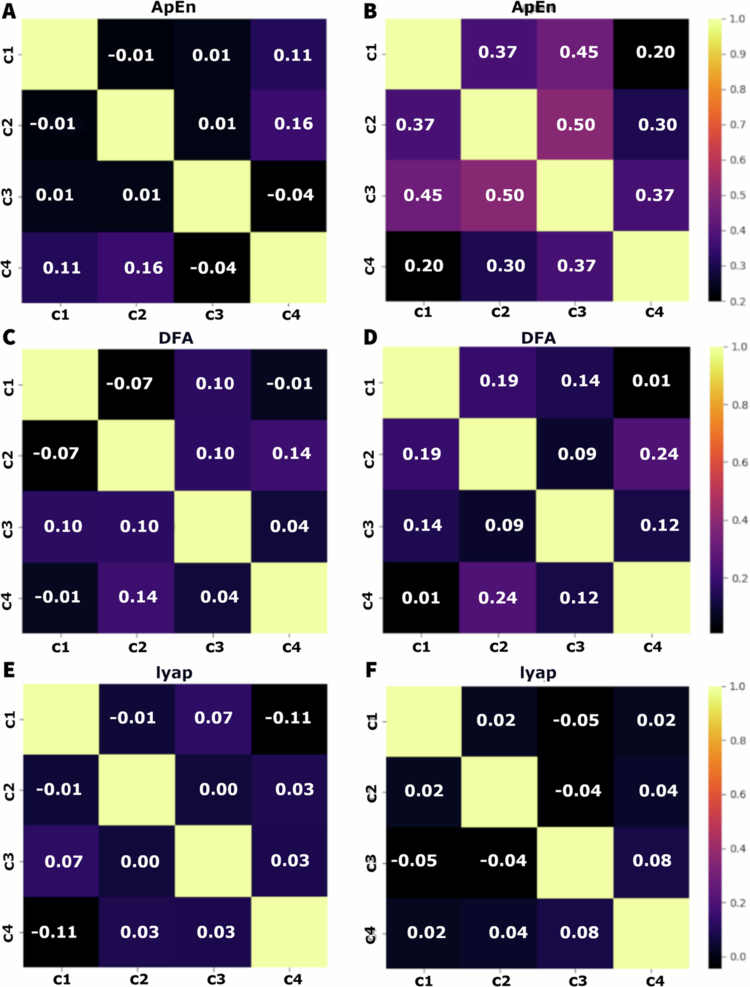
Correlation matrices of time series of approximate entropy (ApEn) (A, B), detrended fluctuation analysis (DFA) (C, D), and Lyapunov exponent (Lyap) (E, F) before (A, C, and E) and after (B, D, and F) the application of cutting and fire stress. Each matrix displays the correlation coefficients between the different modules: stem, first leaf, second leaf, and third leaf (C1, C2, C3, and C4, respectively) for each analysis method. Correlation values range from −1 (perfect negative correlation) to 1 (perfect positive correlation), with paler colours indicating stronger correlations and darker colours indicating weaker or negative correlations.

Figure 5 shows that, before the infliction of stress, the correlation between the modules measured by the approximate entropy (ApEn) is low, ranging from −0.01 to 0.16. Higher correlation was observed between C1 and C4 (0.11), indicating a slightly positive correlation. Overall, the correlations between the modules were weak, suggesting a relative electrophysiological independence between them. After the stress, there is an increase in the correlations, especially between C2 and C3 (0.50) and C1 and C2 (0.37), indicating a higher dependence between the signals of the modules. To the DFA, the correlation between the modules before the stress is low, varying between −0.07 to 0.14. The higher correlation is between C2 and C4 (0.14), suggesting an interaction slightly positive. Most of the correlations are weak or negative, indicating a low synchronisation between the modules. After the onset of stress, the correlations increased compared to the non–stressed state. The highest correlation was observed between C1 and C2 (0.19), and C2 and C4 (0.24), suggesting an intensification of the interaction between the modules. The correlations to the exponent of Lyapunov before stress are low, varying between −0.11 and 0.07. The highest correlation was observed between C1 and C3 (0.07). Negative correlations, like those between C1 and C4 (−0.11) suggest a possible de–synchronisation between some modules. After the stress, the correlations remain low, but show a slight trend to increase, varying from −0.05 to 0.08.

**Figure 6.**
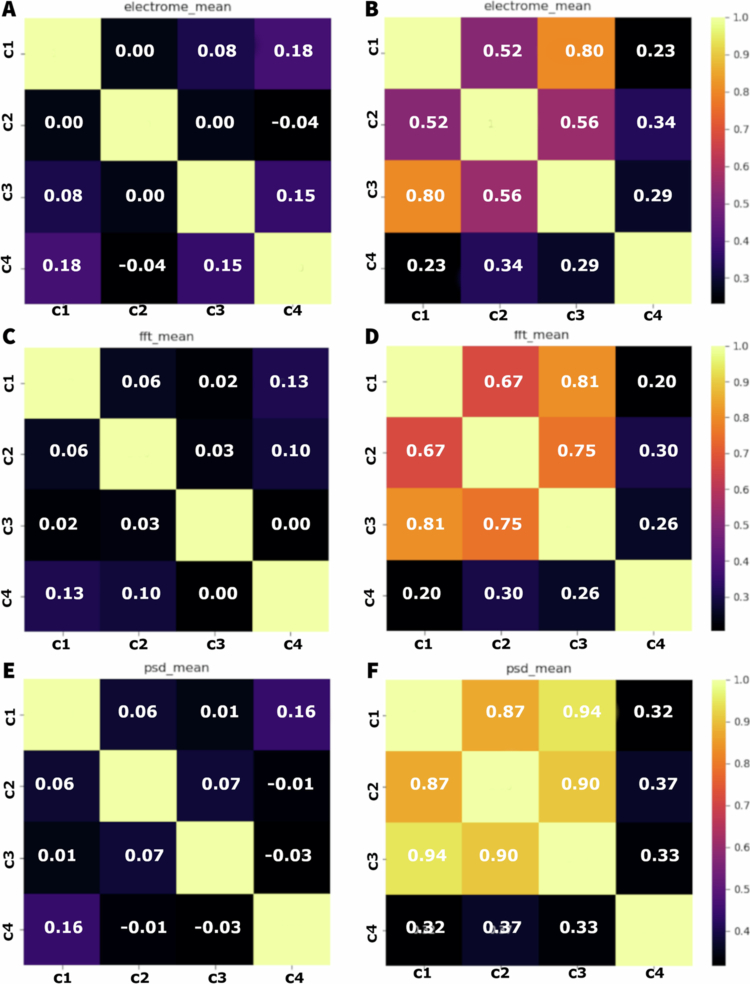
Correlation matrices of the time series for electrome (A, B), FFT (C, D), and PSD (E, F) measurements before (A, C, and E) and after (B, D, and F) the application of cutting and fire stress to the plants. Each matrix displays the correlation coefficients between the different modules: stem (C1), first leaf (C2), second leaf (C3), and third leaf (C4) for each analysis. Correlation values range from −1 (perfect negative correlation) to 1 (perfect positive correlation), with paler colours indicating stronger correlations and darker colours indicating weaker or negative correlations.

For the measures of electrical potential difference before the application of the stress, Figure 6 shows that the correlations between the components are relatively low, varying from −0.04 to 0.18. The highest correlation observed is between C1 and C4 (0.18), indicating a slight positive correlation. Correlations near zero, like the ones between C1 and C2, and C2 and C4, suggest a considerable interdependence between many modules. After the application of the stress, the correlations increased significantly, varying from 0.23 to 0.80. The highest correlation was observed between C1 and C3 (0.80), indicating a strong positive correlation. High correlations are also observed between C1 and C2 (0.52), and C2 and C3 (0.56), suggesting that higher interdependence between the components after stress. For the FFT measures, the correlations before the stress are low, varying from 0.00 to 0.13. Correlations near zero, like the ones between C3 and C4, indicate independence between the modules. After the stress, the correlations increased dramatically, varying from 0.20 to 0.81. The higher correlation is observed between C1 and C2 (0.67) and between C2 and C3 (0.75), highlighting the intensification of the interaction between the modules. For the PSD measures, the correlations before the stress varied from 0.03 to 0.16. The highest correlation was observed between C1 and C4 (0.16), indicating a positive correlation. After the stress, the correlations increased significantly, varying from 0.32 to a remarkable 0.94, the highest value observed. The highest correlation was observed between C1 and C3 (0.94), indicating a relation extremely strong. High correlations were also observed between C1 and C2 (0.87), and between C2 and C3 (0.90).

Overall, before the infliction of cutting and fire, the interaction between the leaves was relatively low, suggesting greater independence of the modules. After the stress, there was a significant increase in synchrony, suggesting a coordinated response to the injury. The leaf directly affected by the injury (C3) shows an increase in the correlation with other modules, especially with the stem (C1) and the first leaf (C2).

### 3.2. Salt–systemic stress

**Figure 7.**
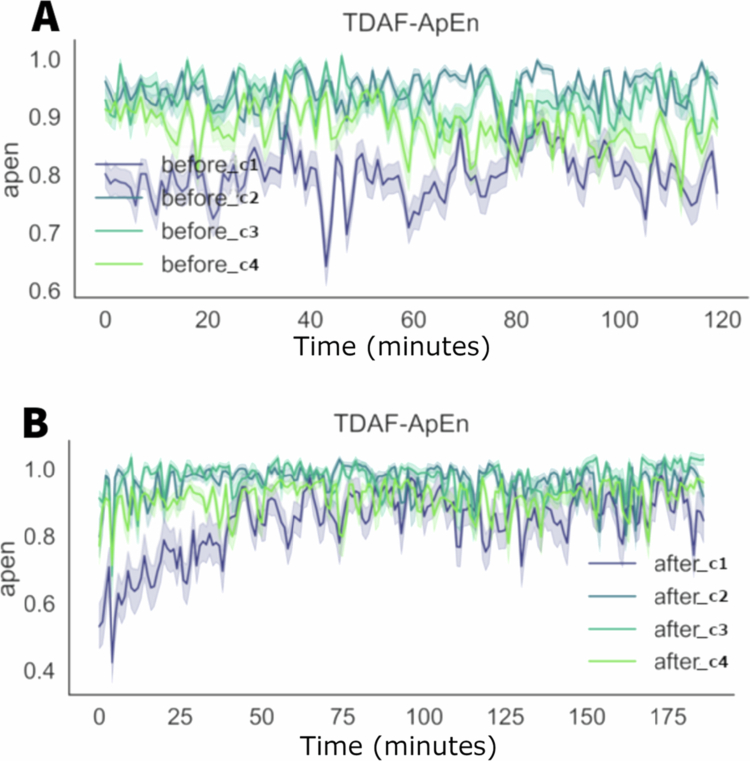
TDAF plot showing the ApEn values. The (A) panel displays measurements taken 120 minutes before the application of salt stress, while the (B) panel shows measurements taken 180 minutes after the stimulus. Dark lines represent the median values at each minute, while shaded areas indicate the maximum and minimum recorded values. The analyses correspond to the following modules: stem (C1), first leaf (C2), second leaf (C3), and third leaf (C4).

In Figure 7, it is possible to observe the values for the approximate entropy (ApEn) before and after the application of the salt solution. Before the application, the bioelectrical behaviour of the stem was different (C1), showing values with low complexity when compared to the leaves. This behaviour suggests a higher variability or irregularity in the data of the stem. On the other hand, the leaves (C2, C3, and C4) show individuality in their patterns of complexity, with ApEn values relatively higher and more variated, indicating higher variability in the signals recorded. After the application of the salt solution, it is observed a tendency of homogenisation of the ApEn between the leaves (C1, C2, and C3). For the stem (C1), there is gradual increase in the complexity, taking up to an hour for the values reach the same of the leaves.

**Figure 8.**
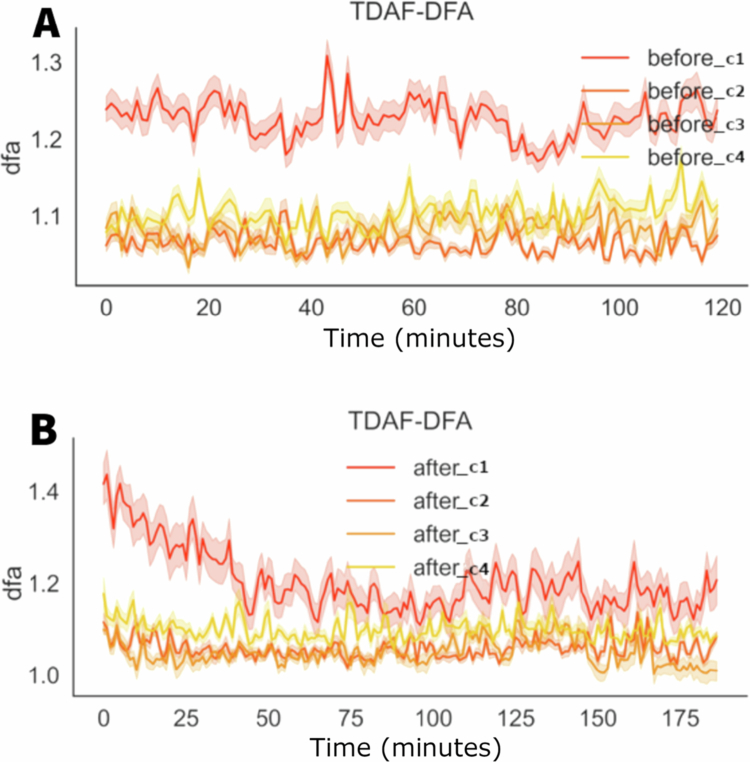
TDAF plot showing the DFA values. The (A) panel displays measurements taken 120 minutes before the application of salt stress, while the (B) panel shows measurements taken 180 minutes after salt stress application. Dark lines represent the median values at each minute, while shaded areas indicate the maximum and minimum recorded values.

Figure 8 shows the values for the detrended fluctuation analysis (DFA) before and after the application of salt stress. Before the stress, the stem (C1) presents the higher DFA values, indicating higher complexity or long–term correlation of the signals in the stem in comparison to the leaves (C2, C3, and C4).

After the application of the salt stress, noticeable changes in the DFA values are observed. This decrease is accompanied by a slight increase in the dispersion of the values in the second leaf (C2), indicating higher individual variability after a stress. The stem (C1) presents higher variation, with a significant reduction in its DFA values throughout the measuring period, with its values approaching those of the leaves.

**Figure 9.**
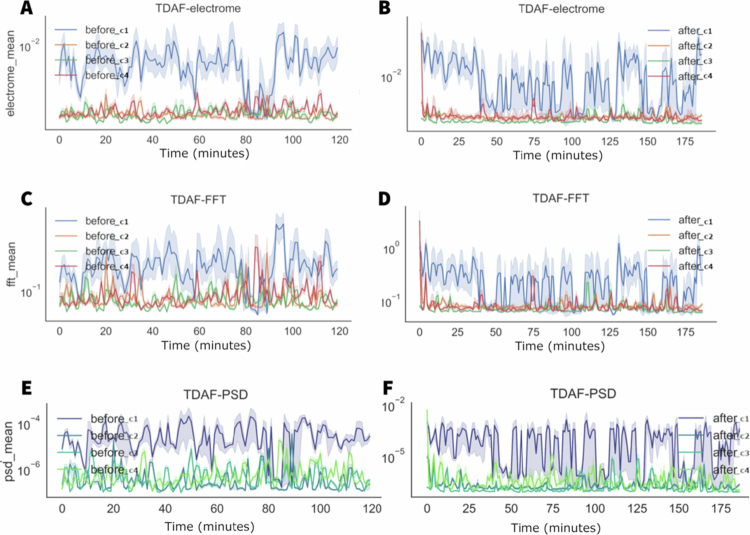
TDAF plot showing the average values for frequency, potential difference, and PSD. The (A) panels display measurements taken 120 minutes before the application of salt stress, while the (B) panels show measurements taken 180 minutes after salt stress application. Dark lines represent the median values at each minute, while shaded areas indicate the minimum and maximum recorded values.

In Figure 9, before the stress, the stem (before1) presented, on average, a higher value of electrical potential difference throughout the measurement period. However, between minutes 80 and 90, there was a decrease in the average values of electrical potential difference for the stem. Nevertheless, there is a significant increase in the dispersion, indicating a higher individuality and variability in the responses of the stem.

The analyses of frequency and energy suggest similar behaviours. Before the stress, a significant difference between leaves and stem is observed, especially during the time between minutes 80 and 90, where the values show higher similarity. After the stress, there is tendency in the stem values to become similar to the leaves. However, this happened for some individuals that indicated this decrease. Other modules in the stem kept presenting higher values, resulting in a higher dispersion of the values for the stem.

**Figure 10.**
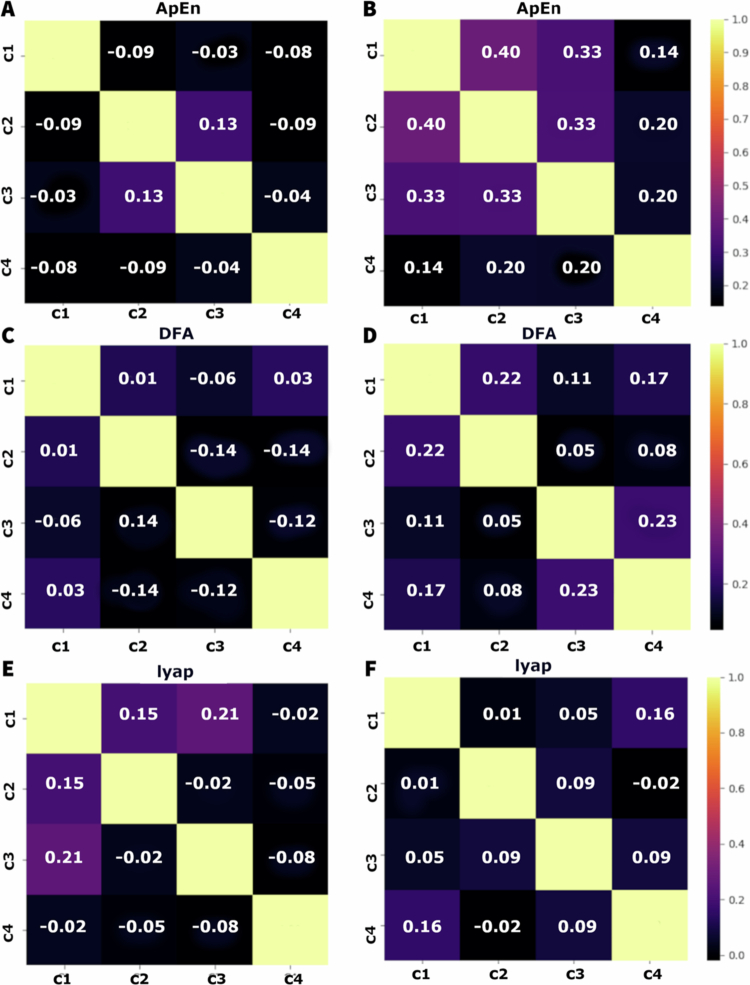
Correlation matrices of the time series for approximate entropy (ApEn) (A, B), detrended fluctuation analysis (DFA) (C, D), and the Lyapunov exponent (Lyap) (E, F) before (A, C, and E) and after (B, D, and F) the application of salt stress. Each matrix displays the correlation coefficients between the different modules: stem, first leaf, second leaf, and third leaf (C1, C2, C3, and C4, respectively) for each analysis method. Correlation values range from −1 (perfect negative correlation) to 1 (perfect positive correlation), with paler colours indicating stronger correlations and darker colours indicating weaker or negative correlations.

The plots presented in Figure 10 show the correlation matrices for three different parameters (ApEn, DFA, and Lyapunov exponent) before and after salt stress. The correlations are represented for 4 experimental conditions: stem (C1), first leaf (C2), second leaf (C3), and third leaf (C4). The colours vary from black to yellow, indicating correlations from −1 to 1, respectively. For the ApEn before the stress, the correlation between the modules is predominantly low or negative. C1 have negative correlations with C2 (−0.09), C3 (−0.03), and C4 (−0.08). C2 and C3 show a low positive correlation (0.13). C4 has slightly negative correlation with the other modules. After the stress, there is an increase in the positive correlations. C1 has positive correlations with C2 (0.40), C3 (0.33), and C4 (0.14). C2 has positive correlations with C3 (0.33) and C4 (0.20). For the DFA before the salt stress, the correlations are also low or slightly negative. C1 has correlations near 0 with C2 (0.01), C3 (−0.06), and C4 (0.03). C2 has negative correlations with C3 (−0.14) and C4 (−0.14). C3 and C4 have negative correlation (−0.12). After the stress, the positive correlations increase slightly. C1 has positive correlations with C2 (0.22), C3 (0.11), and C4 (0.17). C2 has a positive correlation with C3 (0.05) and C4 (0.08). C3 and C5 show a positive correlation of 0.23.

As for the Lyapunov exponent before the stress, the correlations are mixed, with some positive and some negative. C1 has positive correlations with C2 (0.15) and C3 (0.21), and negative correlation with C4 (−0.02). C2 has slightly negative correlations with C3 (−0.02) and C4 (−0.05). C3 and C4 have a negative correlation (−0.08). After the stress, the correlations became more uniform, but still relatively low. C1 has correlations near to zero with C2 (0.01), C3 (0.05), and C4 (0.16). C2 has a slightly positive correlation with C3 (0.09) and a negative correlation with C4 (−0.02). C3 and C4 have a positive correlation of 0.09.

**Figure 11.**
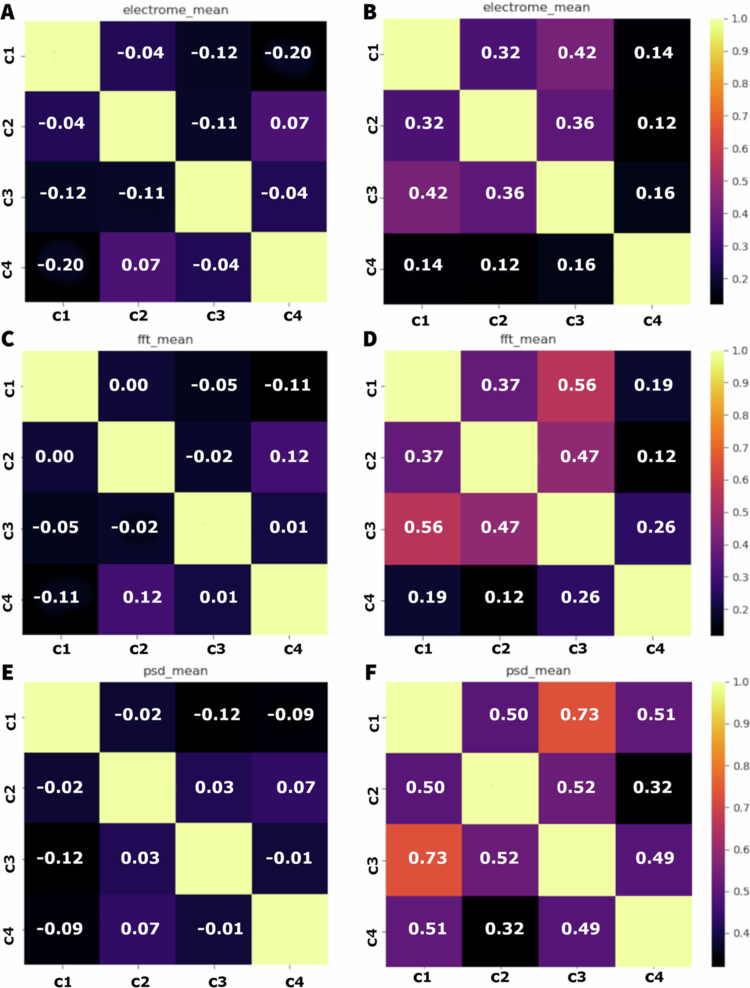
Correlation matrices of the time series for electrome (A, B), FFT (C, D), and PSD (E, F) measurements before (A, C, and E) and after (B, D, and F) the application of salt solution to the plants. Each matrix displays the correlation coefficients between the different modules: stem (C1), first leaf (C2), second leaf (C3), and third leaf (C4) for each analysis. Correlation values range from −1 (perfect negative correlation) to 1 (perfect positive correlation), with paler colours indicating stronger correlations and darker colours indicating weaker or negative correlations.

The plots in Figure 11 show the correlation matrices for three different parameters: the averages of electrical potential difference, frequency, and PSD, both before and after salt stress. The correlations are presented for four experimental conditions: stem (C1), first leaf (C2), second leaf (C3), and third leaf (C4). The colours range from black to yellow, indicating, respectively, correlations from −1 to 1. For the electrical potential difference before the stress, the correlation between C1 and C3 (−0.12) and between C1 and C4 (−0.20) is negative, indicating an inverse relation. The correlations between C2 and C3 (−0.11), and between C3 and C4 (−0.04) are also negative, but of a lower magnitude. The correlation between C2 and C4 (0.07) is slightly positive. After the stress, there is an increase in the correlation between C1 and C2 (0.32), C1 and C4 (0.14), and C1 and C4 (0.14). The correlations between C2 and C3 (0.36), between C2 and C4 (0.12), and between C3 and C4 (0.16) increase slightly.

For the frequency averages before the salt stress, the correlation between C1 and the other modules are near zero, indicating little or no relation. C2 and C3 (−0.02) and C3 and C4 (0.01) show very low correlations. The highest correlation observed is between C2 and C4 (0.12), but this is still relatively low. After the stress, the correlations increased significantly: C1 and C2 (0.37), C1 and C3 (0.56), and C1 and C4 (0.19). The correlations between C2 and C3 (0.47) and between C3 and C4 (0.26) also increased.

For the PSD, before the stress the correlations are predominantly negative or near zero, indicating little or no correlation between the electrical signalling of the modules. C1 and C3 (−0.12) and C1 and C4 (−0.09) have negative correlations. C2 and C3 (0.03) and C2 and C4 (0.07) are slightly positive, but insignificant. After the stress, the correlations increase considerably: C1 and C2 (0.50), C1 and C3 (0.73), and C1 and C4 (0.51).

**Figure 12.**
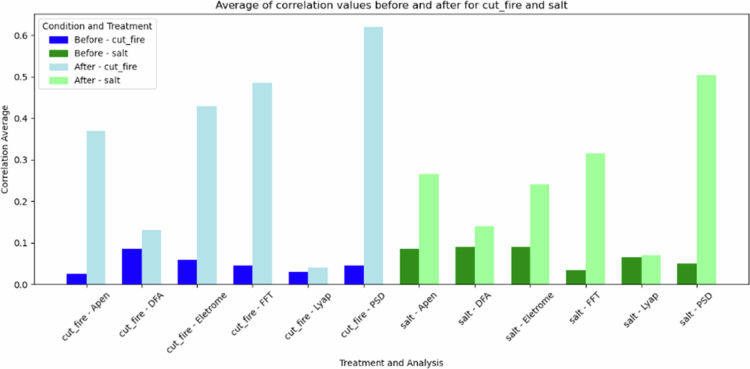
Comparison of the average correlation between the four plant modules (stem, first leaf, second leaf, and third leaf). The blue bars represent the results for cut and fire stress, while the green bars represent the results for salt stress. The bars compare the average correlation before (darker shades) and after (paler shades) the application of stress.

Figure 12 shows the mean average of the correlation found between the modules analysed. Both stresses show a significant increase in the correlation, especially in the PSD, FFT, and electrical potential difference analysed. The cut and fire stress caused higher increases in correlation when compared to salt stress.

## 4. Discussion

This study demonstrates that distinct stress conditions–localised (cutting and fire) and systemic (salinity)—can induce different patterns of electrical synchronisation between modules in soybean plants. These findings reinforce the emerging view that bioelectrical dynamics are integral to whole–plant coordination, likely supporting flexible responses to environmental challenges.^32^

Under baseline conditions, electrical activity across modules exhibited low correlation, suggesting a degree of electrophysiological autonomy among modules. However, after the application of stress, especially cutting and fire, the correlation between modules increased significantly across multiple signal descriptors. This shift suggests a transient functional integration, aligning with the role of electrical signals as rapid long–distance messengers in plants.^6^^,^^33^^,^^34^

While our results demonstrate a strong association between stress and increased electrical synchronisation, we caution against assuming causality. Synchronisation may reflect coordination, but not necessarily a goal–oriented internal state. To test whether synchronisation actively modulates system–wide prioritisation, future experiments should seek causal links. For example, targeted disruption of electrical signalling pathways (e.g., glutamate or mechanosensitive ion channels, or phloem signal blockage) could help determine whether systemic prioritisation of physiological responses is impaired.^17^^,^^35^^,^^36^

The distinct temporal profiles of salt versus mechanical stress responses further suggest stimulus–specific synchronisation dynamics. Salt stress produced slower, more progressive increases in synchronisation, consistent with the need for long–term homoeostatic regulation. In contrast, the rapid integration following cutting/fire reflects a need for fast systemic alerting, a pattern similar to animal systems under threat.^8^^,^^37^^,^^38^

Mechanical injuries, such as cuts, promote the release of chemical mediators as jasmonic acid and glutamate, which activates glutamate receptors (e.g., GLR3.3/3.6) and mechanosensitive channels (e.g., OSCA1.1), triggering Ca²⁺ influx and membrane depolarisations that propagate through the phloem as variation or action potentials.^36^ These electrical signals are accompanied by the local production of reactive oxygen species (ROS) and the activation of signalling pathways such as MAP kinases and cGMP, amplifying the initial chemical signal and promoting the expression of defence genes.^38^^,^^39^ Fire, in turn, causes intense thermal stress, inducing the rapid accumulation of ROS, which not only damage cellular components but also activate heat shock transcription factors (HSFs), responsible for inducing heat shock proteins (HSPs) involved in protection and protein refolding.^40^ In the case of burns, the heat–induced electrical signals can enable the rapid dissemination of information, favouring the physiological adjustment of unaffected parts and contributing to the regeneration of damaged tissues, as the increase in both salicylic and abscisic acid levels in non–damaged distant tissues.^41^

In contrast, salinity stress, as systemic and progressive in nature, led to a gradual, yet significant, synchronisation. Under saline conditions, the plant detects excess Na⁺ in the apoplast, promoting Ca²⁺ influx and activating the SOS (Salt Overly Sensitive) pathway. In this pathway, the SOS3 sensor interacts with the kinase SOS2, which phosphorylates the antiporter SOS1, promoting Na⁺ extrusion and restoring ionic homoeostasis.^6^^,^^42^^,^^43^ Simultaneously, salinity induces the production of ROS, which act as second messengers in the activation of MAP kinases and in regulating the expression of antioxidant and defence genes.^40^ The propagation of electrical signals triggered by such ionic unbalances could enable a coordinated response in different parts of the plant, adjusting physiological processes such as photosynthesis and transpiration to mitigate the effects of salt stress (see some examples in Ref. 44).

These differences in metabolic responses likely reflects the nature of the stimulus, i.e. localised stresses require immediate responses to contain damage, while salinity requires continuous homoeostatic adjustments over time.^43^ In both cases, electrical synchronisation appears to act as an integrative mechanism, likely allowing different modules to coordinate physiological processes such as photosynthesis and transpiration.^40^^,^^44^

Overall, our analyses revealed that different signal complexity metrics–such as approximate entropy (ApEn), detrended fluctuation analysis (DFA), and power spectral density (PSD)—respond differently to stress. Increased ApEn may reflect elevated signal variability, potentially representing greater flexibility or sensitivity to input. DFA convergence between modules may indicate enhanced long–term memory coherence. Higher PSD correlations, in turn, could reflect activation of shared oscillatory components across tissues.^45^ These transitions are reminiscent of the transient synchronisation of neural circuits during attention–like states in animals.^18^^,^^46^

From an ecological and evolutionary standpoint, synchronisation of bioelectrical activity could confer adaptive advantages. Modular plants experience environmental heterogeneity spatially; selective attention–like processes would enable global coordination without unnecessary activation of all tissues. This aligns with the hypothesis that attention, even in basal forms, could improve adaptive responses, prioritising salient stimuli and suppressing less relevant signals.^16^^,^^17^^,^^47^

In conclusion, we propose that increased electrical synchronisation following stress reflects a functional shift toward integrated systemic processing. While speculative, this phenomenon may constitute an attentional–like state, enhancing the efficiency of information processing in a sessile, modular organism. This study lays the groundwork for future mechanistic research into plant attention, cognition, and electrical integration.

## Data Availability

https://1drv.ms/f/c/f5058fece847c076/EnbAR-jsjwUggPWzMQsAAAABMGG5lVbbL0I9Etnd7VKOaA?e=ymv167
